# Genetic alterations and in vivo tumorigenicity of neurospheres derived from an adult glioblastoma

**DOI:** 10.1186/1476-4598-3-25

**Published:** 2004-10-06

**Authors:** Patrizia Tunici, Lorena Bissola, Elena Lualdi, Bianca Pollo, Laura Cajola, Giovanni Broggi, Gabriella Sozzi, Gaetano Finocchiaro

**Affiliations:** 1 Istituto Nazionale Neurologico Besta, Dept. Experimental Neurology, Milano, Italy; 2 Istituto Nazionale Tumori, Dept. Experimental Oncology, Milano, Italy; 3 Istituto Nazionale Neurologico Besta, Dept. Clinical Neurosciences, Milano, Italy; 4 Istituto Nazionale Neurologico Besta, Dept. Neurosurgery; Milano, Italy

## Abstract

Pediatric brain tumors may originate from cells endowed with neural stem/precursor cell properties, growing in vitro as neurospheres. We have found that these cells can also be present in adult brain tumors and form highly infiltrating gliomas in the brain of immunodeficient mice. Neurospheres were grown from three adult brain tumors and two pediatric gliomas. Differentiation of the neurospheres from one adult glioblastoma decreased nestin expression and increased that of glial and neuronal markers. Loss of heterozygosity of 10q and 9p was present in the original glioblastoma, in the neurospheres and in tumors grown into mice, suggesting that *PTEN *and *CDKN2A *alterations are key genetic events in tumor initiating cells with neural precursor properties.

Recent data have proposed that brain tumors contain a "core" of stem cells providing them with the potential to grow aggressively, escaping the effects of radiotherapy and chemotherapy [1,2]. These cancer stem cells were isolated from medulloblastomas or gliomas and grew in vitro as neurospheres, suspended clonal aggregates containing cells with different levels of commitment [3].

Such observations, derived from pediatric tumors only, did not include data on the in vivo tumorigenicity of cancer stem cells. We have found that neurospheres from an adult glioblastoma (GBM) have the potential to express glial and/or neuronal markers and form highly infiltrating gliomas into the brain of immune-deficient mice.

The neurospheres were derived from three adult brain tumors and two pediatric malignant gliomas (BT1–BT5, see Additional file 1). The neurospheres of BT1, a glioblastoma multiforme (GBM) were studied by flow-cytometry and immunohistochemistry. Under differentiating conditions (EGF-bFGF-LIF withdrawal and FBS addition) nestin expression decreased and BT1 neurospheres expressed high levels of neuronal and astrocytic markers. Remarkably, most of the cells expressed both such markers, suggesting the altered function of a complete differentiation program (see Additional file 2).

To test their neoplastic potential we injected BT1 and BT2 (a central neurocytoma) neurospheres into nude mice. All the mice injected intracerebrally (i.c.) with BT1 neurospheres, but none of those injected subcutaneously (s.c.), developed brain tumors that were lethal after 3, 5 and 6 months, respectively. After 4 months, however, none of the mice injected with BT2 neurospheres developed a tumor. Adherent cells from the same two patients were also injected i.c. and s.c. into nude mice. Two of three mice injected i.c. with BT1 adherent cells, but none of those injected with BT2 cells, developed a brain tumor that were lethal 4 and 5 months after injection, respectively.

All the brain tumors in nude mice appeared as large, infiltrating gliomas (Fig. 1A-B) with features of a grade II-III oligoastrocytoma (Fig 1D-E). Both the primary tumor (Fig 1F), and the tumors in nude mice (Fig 1G-H) expressed nestin.

**Figure 1:**
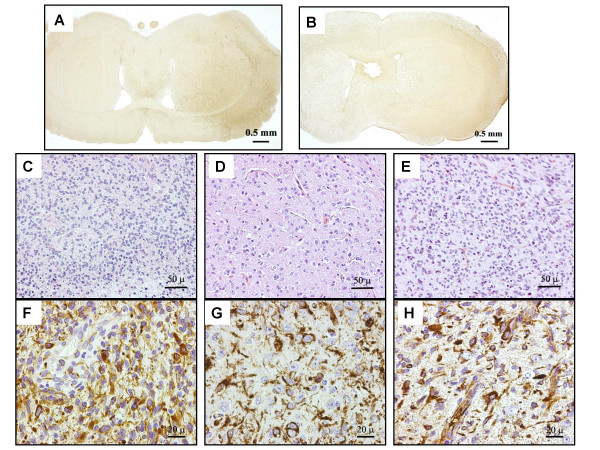
**Histological analysis of BT1 and BT1-derived tumors in nude mice. **BT1 neurospheres (1 × 10e5) were stereotactically injected into the left hemisphere of nude mice (Charles River Italia, Calco, Italy; n = 3) or subcutaneously (n = 3). Nude mice were also injected with 1 × 10e5 BT1 adherent cells into the brain (n = 3) or subcutaneously (n = 3). Cells from BT2 were injected with similar procedures into nude mice. Control mice (n = 3) were injected with 1 × 10e5 neural stem/progenitor cells obtained from C57BL6J mice with previously described methods [11]. Fig 1A-B shows the GFAP staining in brown of coronal sections of the tumor derived from neurospheres (1A) or from adherent cells (1B). The right part on the figures correspond to the left hemisphere, were cells were injected. Fig. 1C-E show H-E staining of the primary tumor with features of a glioblastoma multiforme (1C) and of a tumor in mouse brain derived from neurospheres, showing an area with a prevailing aspect of oligodendroglioma (1D) or adherent cells, exhibiting anaplastic changes (1E). Fig. 1F-H show nestin staining of the primary tumor (1F) and of a tumor in mouse brain derived from neurospheres (1G) or adherent cells (1H).

The five chromosomal regions showing frequent allelic imbalance in gliomas (1p, 9p, 10q, 17p and 19q) were investigated on six specimens obtained from BT1 surgery. No allelic loss was detected in specimen 1 (S1; frontal area); S2 and S3 (fronto-temporal area) showed LOH on chromosome 10q; S4 and S5 (temporal area) had LOH on 10q and 9p (Fig 2). Neurospheres were derived from S6 (temporal) and their analysis showed the same alterations of S5, i.e. LOH on 10q and 9p (Fig. 2). Adherent cells deriving from S6 did not show any detectable LOH and no alteration was found on 1p, 17p and 19q. In the primary tumor the allelic imbalance was partial, in neurospheres, on the contrary, it was complete. Interestingly, not only tumors deriving from BT1 neurospheres but also the tumor from adherent cells showed LOH on 9p and 10q (Fig 2).

**Figure 2:**
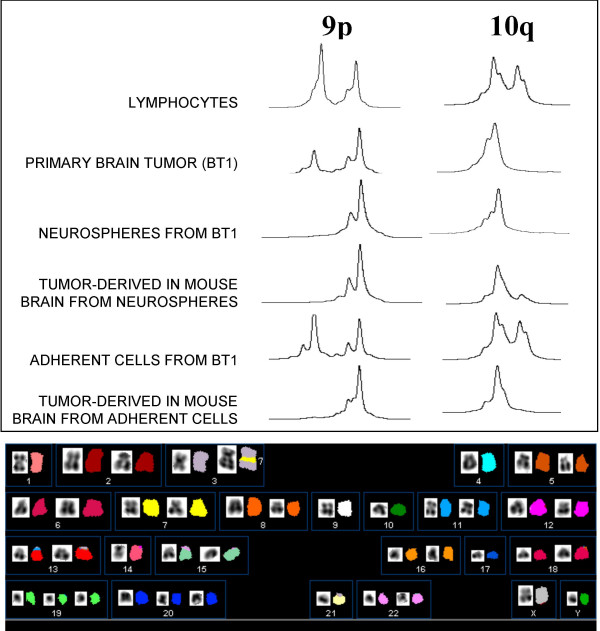
**Genetic analysis on BT1, BT1-neurospheres and adherent cells and BT1-tumors in nude mice. **DNA was extracted from frozen tissues, cell cultures or lymphocytes, using standard protocols. Primers, microsatellite markers and PCR conditions for LOH analysis were described before [12]. We also investigated markers 9S157 and 9S171 flanking the CDKN2A gene on 9p21. Before doing microsatellite analysis on mouse tumors we confirmed that PCR primers did not hybridize on mouse DNA. For cytogenetic analysis cells were harvested with 0.1 μg/ml Colcemid (Karyomax Colcemid, Life Technologies) overnight. Hypotonic treatment, fixation and GTG banding of metaphase chromosomes were performed with standard methods. The karyotypes were described in accordance with ISCN guidelines  Spectral karyotyping was performed on metaphase cells according to the manufacturer's instructions (ASI, Carlsbad, CA) and to published procedures [13]. Spectral images were acquired and analyzed with an SD200 Spectral Bio-imaging System (ASI Ltd., MigdalHaemek, Israel) and a charged-coupled device camera (Hamamatsu, Bridgewater, NJ) connected to a Zeiss Axioskop 2 microscope (Carl Zeiss, Canada) and analyzed by the use of SKYVIEW (version 1.6.1; ASI) software. The *upper panel *shows the results of LOH analysis on 9p and 10q of the different samples outlined on the left. The *lower panel *illustrates a representative spectral karyotype of neurospheres obtained with the simultaneous hybridization of 24 combinatorially labeled chromosome painting probes. Karyotype display of chromosome banding (inverted DAPI) and SKY analysis (chromosomes were assigned a pseudo-color according to the measured spectrum) are shown. The number (7) next to the marker chromosome (der(3)) indicates the origin of inserted material.

Cytogenetic analysis of BT1 neurospheres showed a pseudo-diploid karyotype with monosomy of chromosomes 9, 10, 18, trisomy of chromosomes 19 and 20 and presence of three marker chromosomes. A pseudo-tetraploid clone was also present, resulting from duplication of the pseudo-diploid clone and with the same numerical and structural abnormalities (Fig. 2). The G-banding karyotype of BT1 adherent cells resulted 46, XY. SKY analysis confirmed the numerical changes (monosomies and trisomies) shown by G-banding and allowed to unravel the nature of a the marker chromosomes as a der(3)ins(3;7)(3pter→3q11::7q11→7q22::3q11→3qter).

Three observations are provided by the follow-up of nude mice injected with BT1 cells. First, tumors only developed into the brain and not subcutaneously. Thus, in BT1 the cancer "stem" cells required to be in their niche, i.e. the brain, to develop tumors and the evolution of these tumors resembled closely that of "real" gliomas. The phenotype of such gliomas, however, appeared less aggressive than in the original tumor, possibly because the cancer "stem" cells were conditioned by in vitro passaging and by growth in the brain of immune-deficient mice.

Second, the tumors obtained from neurospheres were completely different from those obtained from established cell lines like U87, 9L, C6 or F98: they grew slower, were highly infiltrating and showed a morphological pattern resembling that of an anaplastic, mixed glioma, but without necrotic areas and palisade cells typical of a GBM (compare Fig. 1C with 1D-E). LOH studies demonstrated the loss of a region chromosome 10q where *PTEN *is located. *PTEN *is a critical tumor suppressor gene in GBM but has also an important role in the regulation of neural stem cell proliferation [4-6]. Its loss can therefore be a central event in the neoplastic derangement of brain cancer "stem" cells. We also found combined 9p LOH associated to 10qLOH in S4–5 and in the neurospheres, but not in S2–3, suggesting that 9p LOH is secondary to that on 10q. LOH on 9p suggests the alteration of the important tumor suppressor gene *CDKN2A*, encoding p16 and p14(ARF). p16 expression is absent or defective in glioblastomas [7,8] and p16 has an important role in the terminal differentiation of neural precursor cells [9]. Furthermore, p16 is the main target through which Bmi1 regulates neural stem cell differentiation and self-renewal [10].

Third, LOH on 10q and 9p were present not only in the original tumor and in neurospheres but also in neurosphere-derived gliomas in nude mice. Remarkably, even if adherent cells had a normal karyotype and no allelic imbalance, the derived tumors did show 10q and 9p LOH. This suggests that few adherent cells with these genetic abnormalities escaped our analysis and underwent a positive selection in vivo. These results, therefore, point to *PTEN *and *CDKN2A *alterations as critical events in tumor initiating cells, a definition synonymous of cancer stem cells.

The identification of neurospheres from adult brain tumors, and specifically from an adult GBM, is strengthening the case for the importance of cancer "stem" cells in the genesis of these malignancies. A thorough genetic dissection of such cells on a larger scale should give new insights for the therapeutic targeting of these cancer "queen-bee" cells.

## Supplementary Material

Additional File 1 Additional file 1 (Tunici et al-Additional file 1.doc) contains Methods with references, comments on in vitro data and the legend to the additional file 2. Click here for file

Additional File 2 Additional file 2 (Tunici et al-Additional file 2.ppt) contains figures of brain tumor neurospheres, and flow cytometry and immunohistochemical data for their characterization. Click here for file
