# Supplementary material for: Genetic alterations and in vivo tumorigenicity of neurospheres derived from an adult glioblastoma
Source: Mol Cancer. 2004 Oct 6;3:25. doi: 10.1186/1476-4598-3-25 (PMC524518; doi:10.1186/1476-4598-3-25)
Supplement: Additional File 1 — (Tunici et al-Additional file 1.doc) contains Methods with references, comments on in vitro data and the legend to the additional file 2. [file 1476-4598-3-25-S1.doc]

ADDITIONAL FILE 1

Brain tumor (BT) neurospheres, prepared as reported by Hemmati et al [1], were obtained after informed consent from three adult and two pediatric patients treated in our Institution: a 36 year-old male with a GBM (BT1), a 54 year-old female with a central neurocytoma (BT2), a 42 year-old male with a relapse for anaplastic astrocytoma (BT3; this is the only tumor previously treated by radiotherapy), a 4 year-old female child with an anaplastic astrocytoma (BT4) and a 8 year-old boy with a GBM (BT5).

Part of BT1 was dissociated and cultured in RPMI and 10% foetal bovine serum (FBS) as adherent cells. Neurospheres from BT1 were analyzed by flow cytometry after exposure to bFGF/EGF/LIF or to 3% FBS for 7 days. Cells were fixed in PBS 1X/ 4% paraformaldehyde, and after several washing incubated with primary antibodies: GFAP (1:200, Dako, Denmark), beta-III tubulin (1:400, Covance, Berkeley, CA), nestin (1:50, Chemicon, Temecula, CA) and CD133 (1:200, Miltenyi Biotec, Auburn, CA). After incubation with FITC- or Cy3-conjugated secondary antibodies (1:300, cells were analyzed by flow cytometry using FACSCalibur (Becton Dickinson, San Jose, CA). Real-time PCR on *BMI1* was performed with the TaqMan assay Hs00180411_m1 (Applied Biosystems, Foster City, CA, USA).

Neurospheres cultured for 7 days with EGF/bFGF/LIF or with 3% FBS were plated onto poly-L-lysine coated chamber slides, fixed in 4% paraformaldehyde, washed with PBS, permeabilized in 0.2% NP-40 and blocked with serum. Cells were incubated with primary antibodies followed by FITC- or Cy3-conjugated secondary antibodies (1:300). Slides were counter-stained with a mounting medium containing DAPI (Vector Laboratories) before examination by fluorescence microscopy.

The primary antibodies were: GFAP (1:200; Dako, Denmark), beta-III tubulin (1:400, Covance, Berkeley, CA), nestin (1:50; Chemicon, Temecula, CA) and CD133 (1:200; Miltenyi Biotec, Auburn, CA).

The proliferation in EGF/bFGF/LIF was steady in all five cases, suggesting that self-renewal and differentiation were taking place at each cell division. Four passages after the plating of 1.5x10e6 cells we obtained after dissociation 3.7 x 10e6 cells from adult BT1 and 7.9 x 10e6 from pediatric BT4. Using a different medium (RPMI with 10% FBS) we could also grow cells as plastic-adherent layers from all tumors (Fig. S1B).

We focused our analysis on BT1 neurospheres. To evaluate their differentiation potential we analyzed by flow cytometry the expression of the stem cell marker nestin [2], and of neuronal and astrocytic markers (beta-III tubulin and GFAP, respectively) under two conditions: presence of EGF/bFGF/LIF; absence of bFG/EGF/LIF and presence of 3% FBS (differentiating conditions). Under differentiating conditions nestin expression decreased from 54.8% to 36.4%, while GFAP and beta III tubulin expression increased, respectively, from 38.5% to 80.1% and from 44.8% to 82% (Fig. S1C-E). Remarkably, a large fraction of cells expressed both the glial and the astrocytic markers as confirmed by immunohistochemistry (Fig. 1G-N). These expression patterns were confirmed by semi-quantitative PCR (data not shown).

The stem cell marker CD133[3], also expressed in the cancer stem cells [1, 4],was expressed in 0.5-1% of BT1 neurospheres, as assessed by flow cytometry (data not shown). This low expression could be explained by the lower abundance of Stem/progenitor cells in adult brain than in newborns or children, as suggested by studies in mice[5].

Expression of Bmi1 a member of the Polycomb family[6],was investigated by real-time PCR in two experiments, and found higher in BT1 neurospheres than in adherent cells (not shown).

Two observations suggest that BT1 neurospheres were not derived by normal stem cells entrapped during tumor growth: i. the microsatellite analysis on 10q and 9p, showing the *complete* absence of the normal allele in the neurospheres as opposed to its *partial* absence in the primary tumor, indicating that all cells in the neurospheres, even if heterogeneous, share the same genetic abnormalities; ii. the lack of neurosphere formation when the starting material derived from the border of a tumor not considered in this study, where normal cells were prevailing (data not shown).

**Figure S1. Isolation and characterization of neurospheres from the BT1 GBM.**

Figure S1 A. BT1 Neurospheres at passage 13 (20X).

Figure S1B. Cell cultured in RPMI with 10% bovine serum grew adherent to the plastic (20X).

Fig. S1C-F. BT1-derived cells were analyzed by flow cytometry as neurospheres (purple peaks) or under differentiating conditions (green peaks) as explained under Methods. Primary antibodies reacted with nestin (S1C), GFAP (S1D), beta III tubulin (S1E) and both GFAP and beta III tubulin (S1F). Percentage represent the mean of two independent experiments.

Fig. S1G-I. Immunohistochemical analysis of BT1 neurospheres after differentiation labeled with antibodies for GFAP (S1G), beta-III tubulin (S1H) and nestin (S1I), all 40X.

Fig. S1L-N show the double labeling of cells fith GFAP (red in S1L) and betaIII-tubulin (green in S1M) and the merging of the two stainings (S1N). All 40X.

REFERENCES

1. Hemmati HD, Nakano I, Lazareff JA, Masterman-Smith M, Geschwind DH, Bronner-Fraser M, Kornblum HI: **Cancerous stem cells can arise from pediatric brain tumors**. *Proc Natl Acad Sci U S A* 2003, **100**(25):15178-15183.

2. Lendahl U, Zimmerman LB, McKay RD: **CNS stem cells express a new class of intermediate filament protein**. *Cell* 1990, **60**(4):585-595.

3. Uchida N, Buck DW, He D, Reitsma MJ, Masek M, Phan TV, Tsukamoto AS, Gage FH, Weissman IL: **Direct isolation of human central nervous system stem cells**. *Proc Natl Acad Sci U S A* 2000, **97**(26):14720-14725.

4. Singh SK, Clarke ID, Terasaki M, Bonn VE, Hawkins C, Squire J, Dirks PB: **Identification of a cancer stem cell in human brain tumors**. *Cancer Res* 2003, **63**(18):5821-5828.

5. Maslov AY, Barone TA, Plunkett RJ, Pruitt SC: **Neural stem cell detection, characterization, and age-related changes in the subventricular zone of mice**. *J Neurosci* 2004, **24**(7):1726-1733.

6. Molofsky AV, Pardal R, Iwashita T, Park IK, Clarke MF, Morrison SJ: **Bmi-1 dependence distinguishes neural stem cell self-renewal from progenitor proliferation**. *Nature* 2003, **425**(6961):962-967.
