# Supplementary figures and images for: Genetic alterations and in vivo tumorigenicity of neurospheres derived from an adult glioblastoma
Source: Mol Cancer. 2004 Oct 6;3:25. doi: 10.1186/1476-4598-3-25 (PMC524518; doi:10.1186/1476-4598-3-25)

## Slide 1
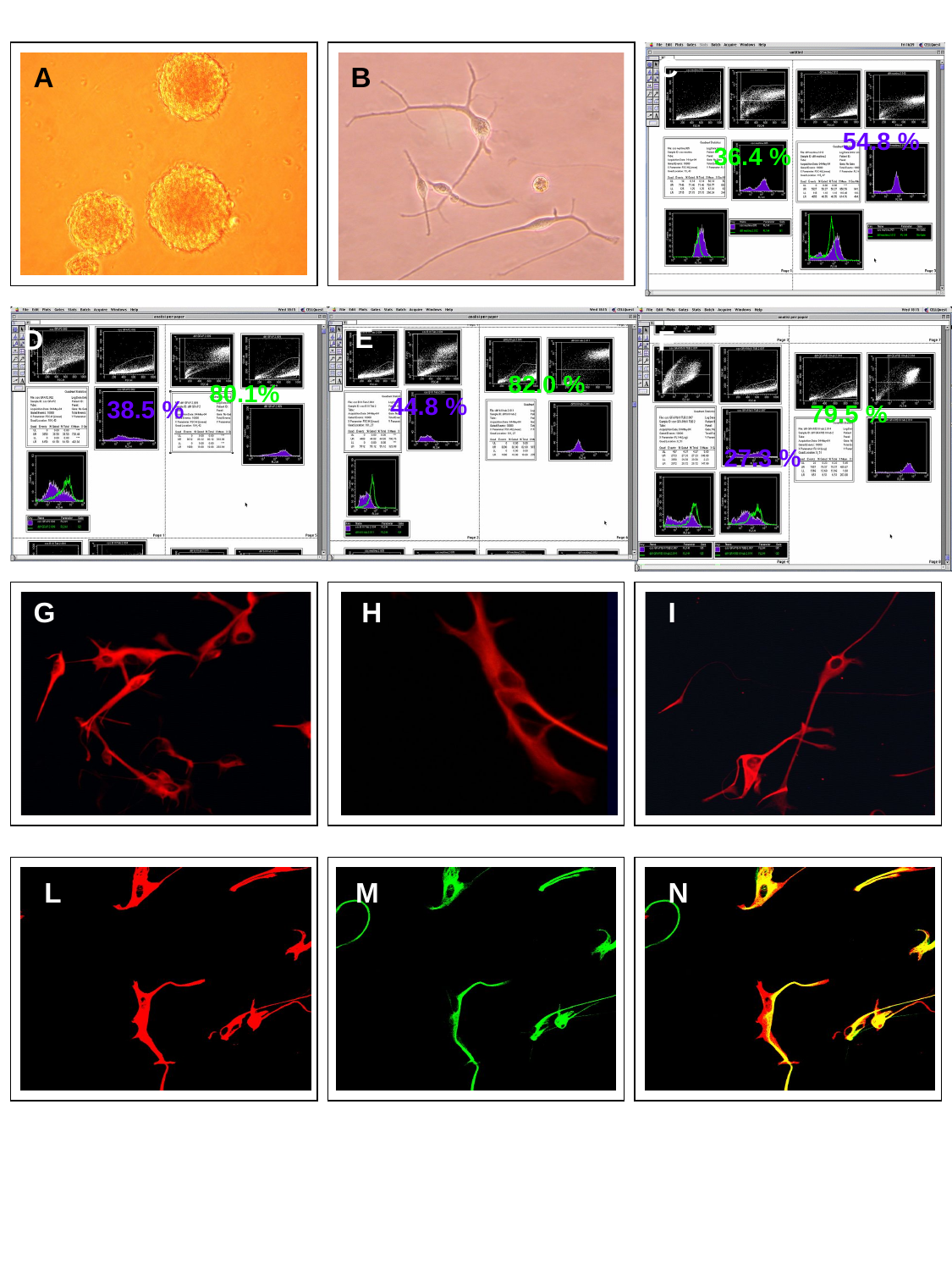

A
B
54.8 %
36.4 %
C
D
80.1%
38.5 %
E
82.0 %
44.8 %
F
79.5 %
27.3 %
G
H
I
L
M
N

Supplement: Additional File 2 — (Tunici et al-Additional file 2.ppt) contains figures of brain tumor neurospheres, and flow cytometry and immunohistochemical data for their characterization. [file 1476-4598-3-25-S2.ppt]
